# Study on the pathogenesis of varicocele induced by the ferroptosis of cremaster satellite cells with the m6A modification of TFRC mRNA

**DOI:** 10.1371/journal.pone.0330666

**Published:** 2025-09-12

**Authors:** Zhiqiang Mo, Weiping Zhang, Xianghui Xie, Ning Sun, Jun Tian, Minglei Li, Hongcheng Song

**Affiliations:** 1 Department of Urology, Shunyi Maternal and Children’s Hospital of Beijing Children’s Hospital, Beijing, China; 2 Department of Urology, Beijing Children’s Hospital, Capital Medical University, National Center for Children’s Health, Beijing, China; Gifu University School of Medicine Graduate School of Medicine: Gifu Daigaku Igakubu Daigakuin Igakukei Kenkyuka, JAPAN

## Abstract

**Background:**

Varicocele (VC) is a leading cause of male infertility. Insufficient growth and development of the cremaster muscle may contribute to VC, but the underlying mechanism remains unclear. Cremaster muscle dysfunction may impair venous valve support, contributing to VC. The cremaster relies on satellite cells (SCs) for postnatal growth and damage repair. This study aimed to explore the mechanism of the cremaster muscle in the process of VC.

**Methods:**

Ten male Sprague-Dawley (SD) rats were divided into two groups: the VC model group (5 rats) and the sham-control group (5 rats). After four weeks of observation, the cremaster muscles were collected. The diameters of the left and right spermatic veins were measured, and the left testis was isolated for morphological examination via H&E staining. SCs isolated from the left cremaster muscle were analyzed using multiple methods, including qPCR and Western blot. Data were analyzed using SPSS v.22.0.

**Results:**

Compared to the control group, the model group showed decreased TFRC mRNA stability, decreased mitochondrial membrane potential, and decreased GSH and GSSG contents, as well as increased m6A modification levels and increased ROS, MDA, and Fe^2+^ contents. In addition, the model group also showed downregulation of transferrin receptor (TFRC, a key iron uptake protein involved in ferroptosis) expression and upregulated m6A methyltransferase and recognition proteins. Multiple biochemical test results indicated increased ferroptosis, characterized by changes such as decreased mitochondrial membrane potential and GSH and increased ROS, MDA, and Fe^2+^.

**Conclusion:**

This study suggests that SCs in the cremaster muscle is associated with impaired cremaster muscle repair and VC pathogenesis through m6A modification of TFRC mRNA. Our findings offer fresh insights into the role of cremaster SCs in VC and provide a foundation for future research on the potential therapeutic target of VC.

**Strengths and limitations of this study:**

This study is the first to investigate the pathogenesis of varicocele from the perspective of the cremaster muscle, and some clues have been discovered from it. The causal relationship between m6A-TFRC axis and ferroptosis requires further validation using functional rescue experiments (e.g., METTL3 knockdown or ferroptosis inhibitors). The small sample size may limit statistical power; future studies with larger cohorts are warranted.

## Introduction

Around the world, 8% ~ 12% of couples suffer from infertility, among whom about 50% are attributed to male infertility [1]. Varicoceles (VC) are a common genitourinary disease in men and a leading cause of male infertility. The incidence of VC is about 15%, and it mainly occurs on the left side [2], accounting for 35% ~ 40% of male infertility [3]. VC refers to the dilation, tortuosity, and lengthening of the spermatic vein plexus, which leads to blood-testis reflux and poor nutrient metabolism, thus causing testicular atrophy, spermatogenesis disorders, and male infertility [4]. The cremaster relies on satellite cells (SCs) for postnatal growth and damage repair.

At present, microscopic varicocele ligation is the gold standard surgical method for VC treatment. Compared with other surgical procedures, microscopic varicocele ligation has a lower risk of postoperative complications, such as testicular atrophy and recurrence [5,6]. However, the probability of natural pregnancy after surgery is still low [5,6]. In general, there is a lack of effective treatment for VC that can improve the semen quality and natural pregnancy rate with fewer complications and lower recurrence.

The limitation of VC treatment may stem from the complex and unknown mechanism of VC. Although many studies have explored the mechanism of male infertility caused by VC, there is a lack of research on the mechanism of VC itself. Studies have shown that the insufficient growth and development of the cremaster muscle may lead to VC, but the underlying mechanism remains unknown. Exploring the molecular mechanism of cremaster muscle in VC may help to find the potential therapeutic target of VC. Recent studies indicate that ferroptosis dysregulation contributes to muscle disorders [7,8], and m6A modification modulates iron metabolism genes [8,9]. However, their roles in cremaster muscle pathology and VC pathogenesis remain unexplored. Our study bridges this gap by investigating TFRC-mediated ferroptosis in SCs as a novel mechanism for VC development. This study aimed to investigate the internal mechanism of the growth and development of the cremaster muscle in the process of VC to guide further therapeutic intervention.

## Materials and methods

### Study subjects and grouping

Ten SPF grade SD rats, 6 ~ 8 weeks old, weighing 200 ~ 220 g, were purchased and housed in the experimental animal center of Beijing Jinglai Huake Biotechnology Co., Ltd. The center was set at a temperature of 24 ± 2 °C and humidity of 50% ~ 70%. Free drinking and eating, as well as regular cleaning and disinfection, were provided. Male Sprague-Dawley rats (6–8 weeks old) were purchased from Jinglai Huake Biotechnology Co., Ltd, Beijing, China and housed in our institution’s specific pathogen-free (SPF) facility. All procedures were approved by the Institutional Animal Care and Use Committee (IACUC) of Jinglai Huake Biotechnology Co., Ltd (Protocol No. SYXK(Jing)-MZQ-2023001).

The ten rats were divided into two groups: the VC rat model (5 rats) and the sham control (5 rats) [1,2]. After the rats were anesthetized, a median incision was made in the abdomen to fully expose and separate the left renal vein segment between the adrenal vein and the inferior vena cava on the left retroperitoneal. A metal rod with a diameter of about 0.6 mm was placed parallel to the left renal vein segment and ligated with 4−0 silk thread. After apparent left renal congestion was observed, the metal rod was then pulled out. When the left renal vein dilation and congestion disappeared, the diameter of the left renal vein ligation was halved. At about four weeks, there was no significant difference in the size and weight between the left and right kidneys. An obvious left VC indicates successful VC modeling. VC modeling success was defined as >50% increase in spermatic vein diameter versus sham controls (measured by ocular micrometer).The sham group received the same treatment as the VC group except for no ligation.Sample size (n = 5 per group) was determined based on prior rodent studies of VC models [2,10] and pilot data showing significant effect sizes (Cohen’s d > 1.5) in key endpoints (e.g., TFRC expression).

Rats were anesthetized via intraperitoneal injection of sodium pentobarbital (40 mg/kg body weight). Depth of anesthesia was confirmed by absence of pedal reflex.

Postoperative analgesia (buprenorphine, 0.05 mg/kg) was administered every 12 hours for 48 hours.After experimental endpoints, rats were euthanized under deep anesthesia (sodium pentobarbital, 80 mg/kg) by cervical dislocation. This method was selected to minimize suffering and comply with AVMA guidelines for humane euthanasia.

### Detection indicators and methods

First, the pathological indicators related to VC were detected as follows:

(1) The anesthetized rats underwent laparotomy, and their left and right spermatic vein diameters were measured using an operating microscope with an eyepiece micrometer.
(2) After the left testis was isolated, the morphological changes were observed by H&E staining, and spermatogenic function was evaluated according to Johnsen’s score.

Then, the left cremaster muscle was isolated and tested as follows:

(3) H&E staining was used to observe the damage.
(4) ROS content was detected by the kit.
(5) The number of SCs in different states was detected by immunofluorescence.

Finally, SCs were isolated from the left cremaster muscle and detected as follows:

(6) The expression level of TFRC was detected by qPCR and Western blot.
(7) The m6A modification level of TFRC mRNA was detected by MERIP-qPCR.
(8) The expression levels of m6A modification key enzymes were detected by qPCR and Western blot.
(9) The morphology of iron death ferroptosis was observed by transmission electron microscopy, including cell membrane, mitochondrial outer membrane rupture, mitochondrial size, mitochondrial membrane density, and other changes.
(10) The expression levels of GPX4 and FTH1 and indicators of ferroptosis were detected by qPCR and Western Blot.
(11) The content of MDA, Fe^2+^, GSH, and GSSG were detected by corresponding kits.

### Statistical analysis

SPSS v.22.0 was used for data analysis. Categorical variables were expressed by numbers and rates, and continuous variables were described by means ± standard deviations. The differences in continuous variables between the two groups were analyzed using a t-test for normally distributed variables and a non-parametric test for non-normally distributed variables. Data normality was assessed by Shapiro-Wilk test before selecting parametric or non-parametric tests.The differences in categorical variables between the two groups were analyzed using Fisher’s exact test or chi-square test.

## Results

Compared to the control group, the model group showed the following differences: TFRC protein decreased by 40% in VC vs. CON (p = 0.001, Fig 4); Mitochondrial potential (JC-1 aggregate/monomer ratio) dropped 2.5-fold (p < 0.001, Fig 3);m6A methyltransferase and recognition protein were upregulated((METTL3 ↑ 1.8-fold, METTL14 ↑ 2.1-fold; p < 0.01) and readers (YTHDF1 ↑ 1.5-fold, YTHDF3 ↑ 1.7-fold; p < 0.05)); TFRC expression was downregulated; overall m6A modification levels increased; the mitochondrial membrane potential decreased; ROS content increased; MDA content increased; Fe^2+^ content increased; GSH content decreased; GSH/GSSG ratio (indicating impaired antioxidant capacity) Multiple biochemical test results showed decreased mitochondrial membrane potential and GSH, as well as increased ROS, MDA, and Fe^2+^, suggesting increased ferroptosis (–7).

**Fig 1:**
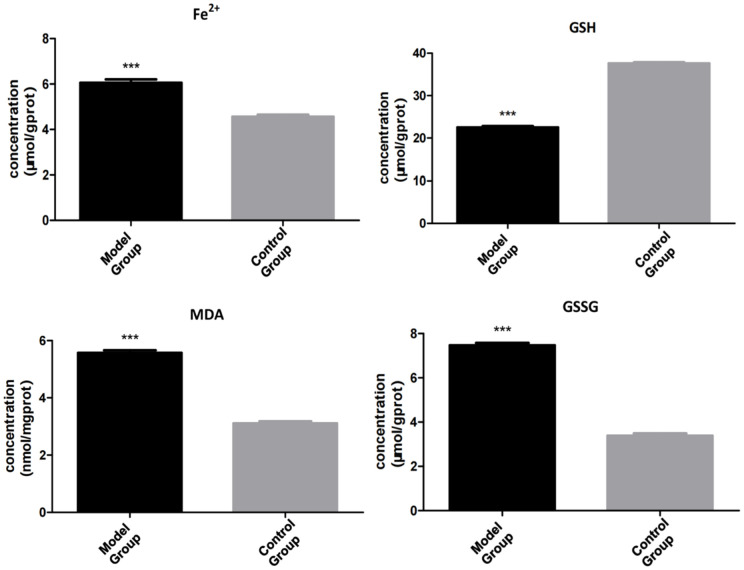
Biochemical indicators of ferroptosis in cremaster muscle. Model Group: varicocele model,Control Group:control, *p < 0.05. Biochemical test results: compared with the control group, the model group had decreased GSSG content of the cremaster muscle, increased Fe^2+^ content, increased MDA content, and GSH/GSSG ratio (indicating impaired antioxidant capacity), suggesting the occurrence of the ferroptosis process.

**Fig 2:**
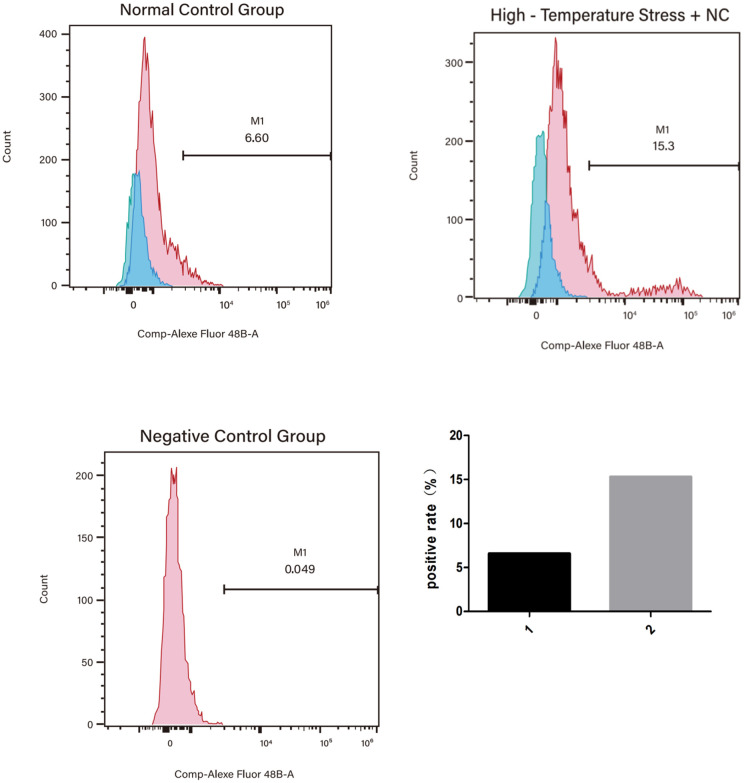
ROS levels in satellite cells. Flow ROS detection: sample 1 was a standard control, and sample 2 was a negative control with high-temperature stress +nc. The results showed that ROS content increased, suggesting the occurrence of the ferroptosis process:ROS levels significantly increased in VC model SCs (p < 0.01).

**Fig 3:**
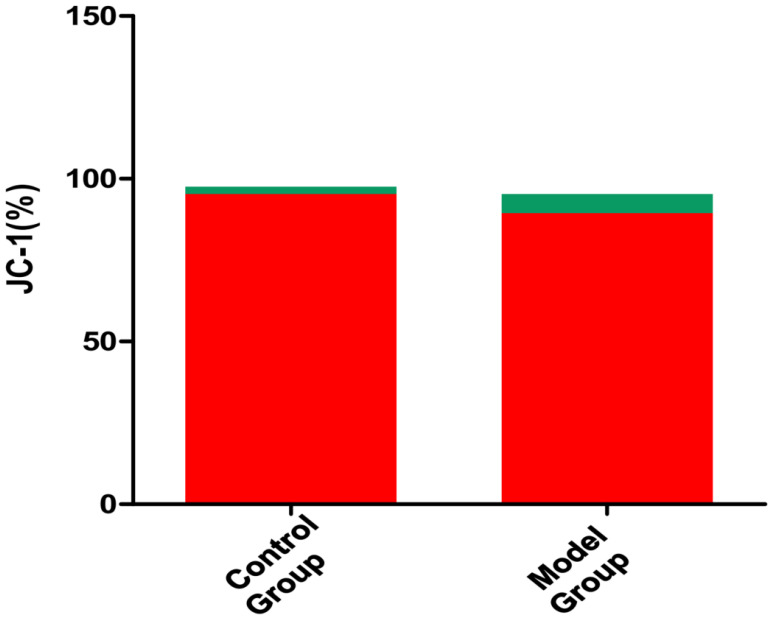
Mitochondrial membrane potential detected by JC-1 staining. Model Group: varicocele model, Control Group: control. Flow cytometry JC-1 detection: when the mitochondrial membrane potential is high, JC-1 accumulates in the matrix of mitochondria to form polymers, which produces red fluorescence. When the mitochondrial membrane potential is low, JC-1 cannot aggregate in the matrix of mitochondria. At this time, JC-1 is a monomer and produces green fluorescence: Mitochondrial membrane potential was significantly reduced in VC group (p < 0.001).

**Fig 4:**
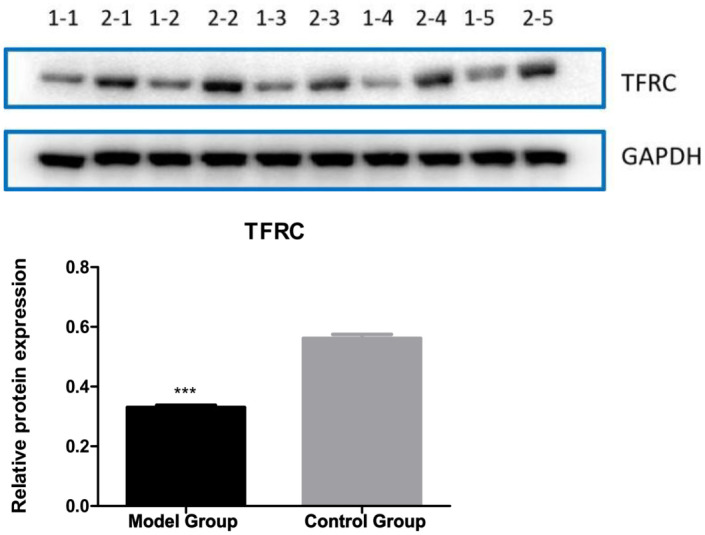
Protein expression of TFRC. Model Group: varicocele model, Control Group: control, *p < 0.05. WB experiment: t-test*, vs. normal control group. 1−1 and 2−1 represent model group 1, control group 1, and so on. The results showed that TFRC expression was downregulated in the model group, suggesting that ferroptosis was increased in the model group.

**Fig 5:**
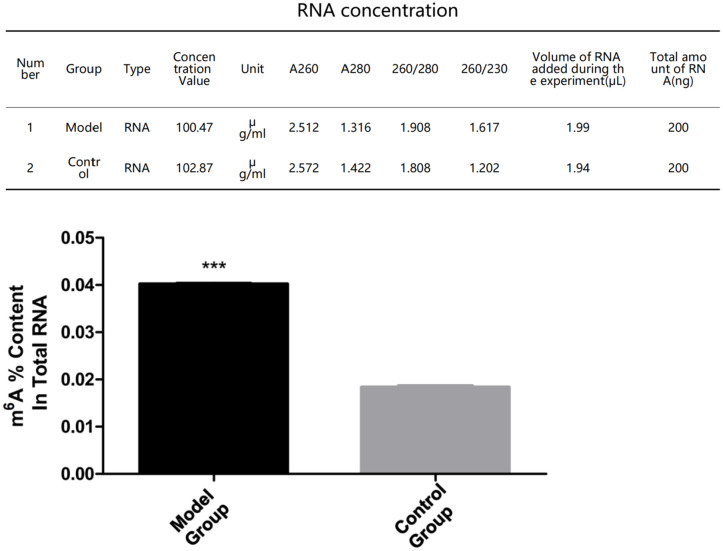
m6A modification level of TFRC mRNA. Model Group: varicocele model,Control Group:control, *p < 0.05.m6A modification level detection of TFRC mRNA: t-test*, vs. normal control group. The results showed an overall increase in the overall m6A modification level:Overall m6A modification level increased by 2.3-fold in VC group (p < 0.01).

**Fig 6:**
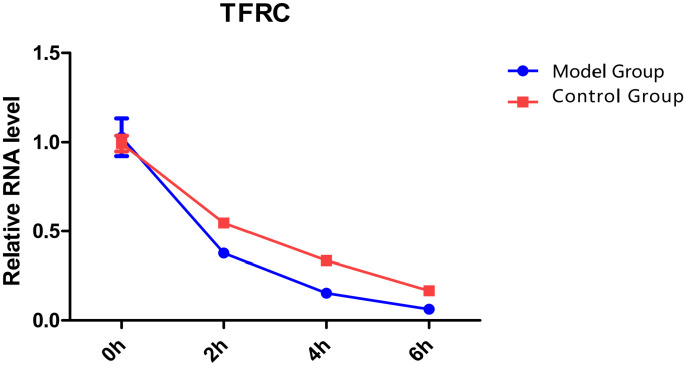
Stability of TFRC mRNA. Model Group: varicocele model, Control Group: control, *p < 0.05. Stability test of TFRC mRNA: t-test*, the results showed that the stability of TFRC mRNA in the model group decreased: TFRC mRNA half-life decreased by 40% in VC group (p < 0.05).

**Fig 7:**
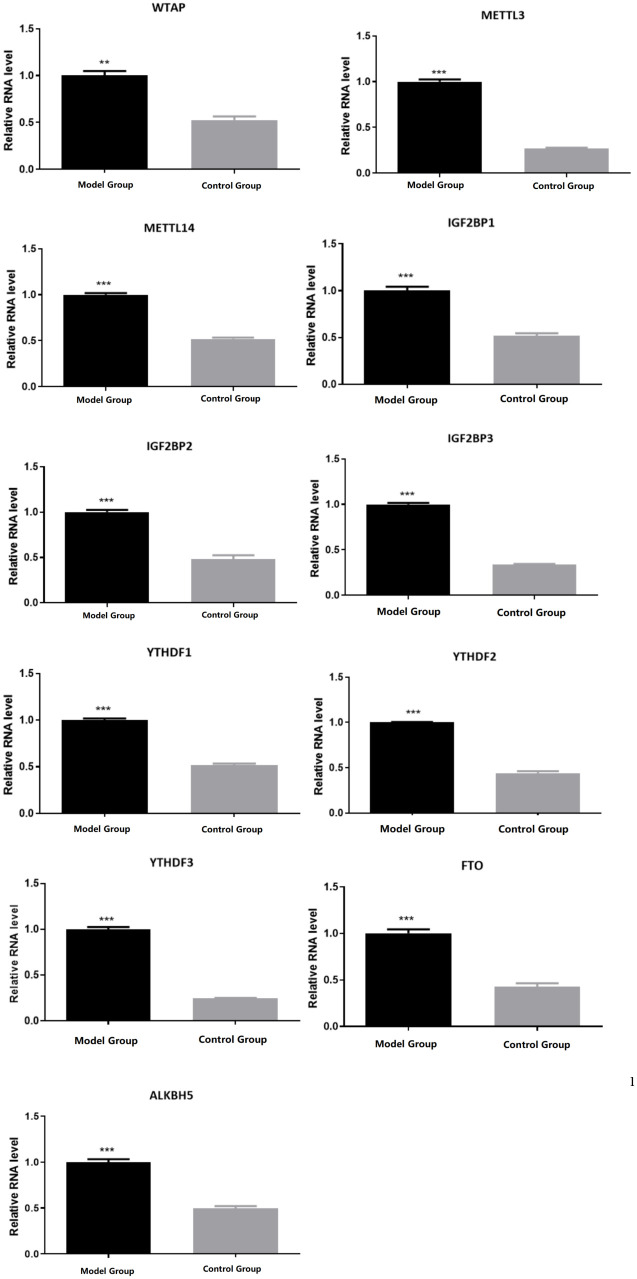
Expression levels of m6A modification regulators. Model Group: varicocele model,Control Group:control, *p < 0.05.The expression levels of m6A modification key genes (including writers: WTAP, METTL3, METTL14, readers: IGF2 BP1/2/3, YTHDF1/2/3, erasers: FTO, ALKBH5) were detected. The results showed that the expression levels of writers (WTAP, METTL3, METTL14), readers (IGF2 BP1/2/3, YTHDF1/2/3), and erasers (FTO, ALKBH5) were upregulated:Key m6A writers/readers were significantly upregulated in VC group (p < 0.05).

TFRC downregulation likely impairs iron homeostasis, accelerating lipid peroxidation in SCs. H&E staining revealed cremaster muscle atrophy and 40% reduction in SC count (p = 0.003). Johnsen scores decreased by 30% (12.3 ± 0.8 vs 8.6 ± 1.1, p = 0.002), correlating with SC loss (r = 0.82, p < 0.05).

## Discussion

### Decreased number of satellite cells

Clinically, the growth and development of the cremaster muscle may be an important reason for the occurrence of VC. However, the intrinsic mechanism has not been revealed so far. Due to the skeletal muscle nature of cremaster muscle, its regulatory mechanism may be understood with reference to the regulatory mechanism of skeletal muscle growth and development.

Postnatal skeletal muscle growth and development mainly rely on SC-mediated repair of skeletal muscle injury [11]. SCs were first found in frog skeletal muscle by Mauro et al. in 1961. They are undifferentiated muscle precursor cells retained in individual muscle tissue. SCs are located between the basement membrane of muscle fibers and the sarcolemma, similar to the arrangement of satellites of muscle cells, and are thus called satellite cells [12]. SCs play a crucial role in promoting skeletal muscle growth and development, injury repair, and skeletal muscle remodeling after birth. Their quiescent self-renewal and activated proliferation, differentiation, and fusion to form myotubes are key steps in the growth and development of skeletal muscle after birth [13]. Some studies further found that the decline in the number of SCS would inhibit the repair of skeletal muscle injury, thus hindering skeletal muscle growth. For example, lysine deficiency leads to SCS apoptosis by inhibiting the Janus kinase 2-signal transducer and activator of the transcription 3 (jak2-stat3) signaling pathway, resulting in decreased SCs and inhibited skeletal muscle development [14]. Knockdown of the Yin and Yang 1 protein (YY1) gene can prevent the metabolic reprogramming of SCs and inhibit their proliferation, leading to decreased SCs and inhibited muscle repair [15]. Maintaining a certain number of SCs is the premise of skeletal muscle repair after injury, and a decrease in SCs may hinder the growth and development of skeletal muscle.

In previous studies, oxidative damage induced by long-term and high-dose reactive oxygen species (ROS) exposure is an essential cause of skeletal muscle injury [16]. In clinics, the increase of ROS is closely related to the occurrence of VC [10,17]. This suggests that in the process of VC, there may be pathological changes of cremaster muscle injury caused by ROS, which requires SCS to promote injury repair. Therefore, we speculate that when the number of SCS in the cremaster muscle decreases, it may be difficult to repair the damage to the cremaster muscle caused by ROS, ultimately leading to VC.

### Downregulated TFRC expression

Our VC rat model showed that there was a correlation between the ferroptosis level in the cremaster muscle and VC. Specifically, the VC model group showed decreased mitochondrial membrane potential of SCs in the cremaster muscle, increased ROS content, increased MDA content, increased Fe^2+^ content, and decreased GSH compared to the control group. These indicators all suggest the occurrence of ferroptosis. Previous studies have shown the existence of ferroptosis in skeletal muscle SCs [7]. Ferroptosis is a form of programmed cell death driven by iron-dependent lipid peroxidation [18]. Phospholipids containing polyunsaturated fatty acids on the cell membrane are prone to peroxidation under conditions rich in Fe^2+^ and ROS. The accumulation of lipid peroxides eventually destroys the integrity of the cell membrane, resulting in ferroptosis [19]. Glutathione peroxidase 4 (GPX4) and cystine glutamate transport receptor (system XC-) are the main signaling pathways associated with ferroptosis [20]. GPX4 inactivation will lead to the accumulation of lipid peroxides and cause a further increase in ROS to promote ferroptosis [21]. Based on the correlation between VC and ROS and previous data, we speculate that there is a correlation between ferroptosis in the cremaster muscle and VC. ferroptosis in SCs of the cremaster muscle may reduce its number, inhibit its growth and development, and may contribute to the occurrence of VC.

Consistent with Ding et al. [17], our data show TFRC downregulation exacerbates iron overload in the VC rat model, which showed that the downregulation of transferrin receptor (TFRC) expression in the cremaster muscle was associated with VC. Specifically, the VC model group had decreased TFRC protein level of cremaster muscle compared to the control group. After entering the blood circulation, Fe^2+^ will bind to TFRC on the cell membrane surface in the form of transferrin (TF) – bound iron and then be endocytosed and absorbed [22]. Therefore, the lack or downregulation of TFRC tends to inhibit ferroptosis [23,24]. While this suggests a protective role, recent paradoxically report that defects in TFRC or TF are associated with increased ferroptosis. For example, the loss of TFRC in skeletal muscle SCs may contribute the absorption of non-transferrin-bound iron and cause Fe^2+^ accumulation, glutathione metabolism disorder, and lipid peroxidation, inducing skeletal muscle ferroptosis [7]. In our VC model, TFRC deficiency likely disrupts iron homeostasis through two mechanisms: (1) Impaired transferrin-bound iron import causes compensatory uptake of non-transferrin-bound iron (NTBI), leading to labile iron pool overload; (2) Dysregulated iron distribution depletes glutathione reserves and inactivates GPX4, as evidenced by decreased GSH/GSSG ratio and GPX4 expression (Fig 4). This dual dysfunction synergistically accelerates lipid peroxidation cascade. Fe^2+^ accumulation and lipid peroxidation exist in patients with liver cirrhosis, but the expression level of TF is low. Studies showed that mice with liver-specific TF deficiency and high iron diets are prone to liver fibrosis caused by ferroptosis [25]. These studies suggest that the downregulation of TFRC expression may contribute ferroptosis and reduce the number of SCs in the cremaster muscle, inhibiting the growth and development of the cremaster muscle and leading to VC.

### m6A modification

In this study, the sRAMP online tool predicted that there were potential m6A modification sites on the TFRC mRNA sequence. Further pre-experiments carried out in the VC rat model showed that increased m6A modification in the cremaster muscle was associated with VC. Specifically, the model group had increased m6A modification of the cremaster muscle compared to the control group. N6 methyladenine (m6A) modification is the most common and dynamically reversible RNA modification on eukaryotic cell mRNA. m6A modification is mainly affected by methyltransferases like 3 (METTL3), METTL14, and other methyltransferases (Writers), and fat mass and obesity-associated protein (FTO). It is also affected by the regulation of demethylases, such as alkylation repair homologue 5 (ALKBH5), and recognition proteins, such as insulin-like growth factor 2 mRNA binding protein 1/2/3 (IGF2 BP 1/2/3) and YTH N6 methyladenosine RNA binding protein 1/2/3 (YTHDF 1/2/3) [24]. Under the synergistic action of writer, eraser, and reader, m6A modification can play different regulatory roles.

Studies have shown that m6A modification mainly regulates mRNA splicing, enucleation, degradation, translation, and other processes [26] and is a critical way to regulate gene expression [27]. Recent studies have found that m6A modification can reduce the stability of target mRNA, thereby inhibiting gene expression [28,29]. To explore whether increased m6A modification in the cremaster muscle affected the stability of TFRC mRNA, this study compared the stability of TFRC mRNA in the cremaster muscle of rats in the control group and the model group. We found that the model group had decreased stability of TFRC mRNA in the cremaster muscle compared to the control group. Combined with these studies, we speculate that m6A modification of TFRC mRNA may contribute the degradation and downregulation of TFRC expression. Previous studies have shown that m6A modification is a potential mechanism to regulate SCs during skeletal muscle growth and development [30,31]. Moreover, m6A modification plays a vital role in the process of ferroptosis [8]. Therefore, m6A modification may reduce TFRC mRNA stability and downregulate its expression, thereby promoting ferroptosis in cremaster SCs.

While our data (decreased GPX4, mitochondrial collapse, Fe² ⁺ accumulation) align with ferroptosis [18–20], we acknowledge that apoptosis may co-occur. Future studies should employ ferroptosis inhibitors (e.g., ferrostatin-1) and apoptosis markers (e.g., cleaved caspase-3) to dissect the relative contributions of ferroptosis and apoptosis to satellite cell loss, including:

(1) Co-staining for ferroptosis markers (e.g., PTGS2) and apoptosis markers (cleaved caspase-3);
(2) Comparative intervention using ferroptosis inhibitors (e.g., ferrostatin-1) versus apoptosis inhibitors (e.g., Z-VAD-FMK).

## Conclusion

Using the VC rat model, we found that the decreased number of SCs in the cremaster muscle was associated with VC in the rat model. In addition, increased ferroptosis in the cremaster muscle and down-regulation of TFRC expression were associated with VC. Furthermore, increased m6A modification of TFRC mRNA in the cremaster muscle was associated with VC. In conclusion, this study suggests that m6A modification of TFRC mRNA is linked to its degradation and reduce the expression level of TFRC, which in turn stimulates the ferroptosis of SCs in the cremaster muscle, coinciding with a decreased number of SCs. The decreased SCs make it challenging to repair the injury of the cremaster muscle caused by excessive ROS, thus inhibiting the growth and development of the cremaster muscle and suggesting a potential role in VC pathogenesis. Therefore, m6A-mediated TFRC suppression correlates with ferroptosis in SCs, suggesting a possible involvement in VC pathogenesis. Targeting this axis (e.g., METTL3 inhibitors) warrants exploration.
